# Healthier Lives for European Minority Groups: School and Health Care, Lessons from the Roma

**DOI:** 10.3390/ijerph10083089

**Published:** 2013-07-24

**Authors:** Ainhoa Flecha

**Affiliations:** Department of Sociology, Autonomous University of Barcelona, Edifici B, Office B3-185, 08193 Bellaterra, Cerdanyola del Vallès, Barcelona, Spain; E-Mail: ainhoa.flecha@uab.cat; Tel.: +34-93-586-82-04; Fax: +34-93-581-28-27

**Keywords:** health inequalities, socioeconomic effects on health, Roma people, educative participation, interactive groups, family education, vulnerable groups

## Abstract

On average, the Roma in Europe can expect to die 10 years earlier than the rest of the population, given the health conditions they experience. EU-funded research has informed on successful actions (SA) that when implemented among the Roma provide them new forms of educational participation which have a direct impact on improving their health status, regardless of their educational level. The findings from this research, unanimously endorsed by the European Parliament, have been included in several European Union recommendations and resolutions as part of the EU strategy on Roma inclusion. To analyze these SA, as well as the conditions that promote them and their impact on reducing health inequalities, communicative fieldwork has been conducted with Roma people from a deprived neighbourhood in the South of Spain, who are participating in the previously identified SA. The analysis reveals that these SA enable Roma people to reinforce and enrich specific strategies like improving family cohesion and strengthening their identity, which allow them to improve their overall health. These findings may inform public policies to improve the health condition of the Roma and other vulnerable groups, one goal of the Europe 2020 strategy for a healthier Europe.

## 1. Introduction

Given the inequalities in health status that continue to prevail in European societies, it remains crucial to develop effective policies in response. People in lower socioeconomic groups die at younger ages and suffer from a higher prevalence of health problems such as chronic conditions, mental health problems, and disabilities [1]. In Europe, two types of health disparities create concern: the disparities among social groups and those among member states. For instance, a 2010 report from the World Health Organization (WHO) compared the life expectancy rates of European member states that joined the EU before and after 2004; the report discovered that for those joining after 2004, the life expectancy was 5.6 years lower [2]. This alarming situation has made improved health a key priority of the Europe 2020 strategy to promote at smart and inclusive growth. In the inclusive growth goal, the EC highlights the need to develop a major effort “to combat poverty and social exclusion and reduce health inequalities to ensure that everybody can benefit from growth” [3]. 

Health disparities are particularly worrisome among vulnerable and socially excluded groups such as the Roma who have been the focus of our research. As various international institutions have reported, it is crucial to acknowledge the blatant exclusion that the Roma people experience within the European Union, particularly regarding health. Specifically, their estimated life expectancy is ten years shorter than that of the general population [4]. The European Commission and the WHO report that this statistic is directly related to the exclusion of the Roma from society [5].

Researchers have traced the health disparities among different social groups to a range of socioeconomic factors including differences in educational level attained, occupational class, and income; gender and race also play significant roles [6]. The associations between these factors are also important; for instance, low income affects educational attainment [7]. In addition, inadequate housing, poor nutrition and health-related behaviours [8], and other factors such as social ties in the local community [9,10] are all powerful determinants of the poor health conditions that affect millions of people around the globe. The WHO’s Regional Office for Europe, in its first *Report on the Social Determinants of Health and the Health Divide* discusses the most relevant social determinants that impact individuals’ health: childhood living conditions, education, working conditions and employment, welfare and social protection, poverty, communities, and health systems. 

The WHO has repeatedly noted that the rates of infant mortality are higher among the children of mothers with no education, especially in certain countries. For such women in Bolivia, for instance, the rate is greater than 100 deaths per 1,000 live births [11]. A 1999 WHO report stated that the poorest and least educated people not only live shorter lives but also are more likely to suffer from disabilities [12]. In particular, the educational attainment of mothers has been found to determine child mortality rates—making female education an urgent focus in reducing infant mortality [13]. 

Available health-related data on rates of premature mortality show that the rates are significantly higher among people with lower levels of education, lower occupational classes, and lower income [1]. Thus, educational level is a key factor influencing health conditions. International institutions such as the WHO and the OECD have provided evidence for this direct relationship [11,14,15]. Indeed, “education is positively associated with a variety of social outcomes, such as better health” [16]. 

### 1.1. Health among the Roma People

The European Union acknowledges that the Roma people are one of the most marginalised social groups, “facing deep social problems related to low levels of education, high unemployment, inadequate housing, poor health, and wide-ranging discrimination” [17]. In particular, they face serious health situations because of their disadvantaged social position. In addition, many researchers note that few rigorous systematic data are available on the Roma community’s health condition [18,19,20]. The data on Roma childrens’ health statuses are inconsistent, due to the challenges of monitoring ethnic groups in Europe, but fragmented evidence indicates high levels of maternal and child mortality and morbidity [5,21]. Furthermore, data from the European Survey on Health and the Roma Community [22] show a high dependency rate among the Roma in Europe: 62 not economically active people for every 100 active ones, as opposed to 48.7 for the EU27. 

Moreover, the available data support the fact that the socioeconomic situation of the Roma affects their health conditions. Along this line, the UNDP [23] report that hunger is a problem in the poorest Roma communities. Of the Roma surveyed by the UNDP in south-eastern Europe, 53% reported going hungry in the previous month, compared with 7% of the non-Roma living in close proximity to these Roma settlements.

The Roma population has also shown a high prevalence of chronic diseases such as migraines, hypertension, and arthritis; dental problems and difficulties with vision and hearing are also widely reported. Again, the European Survey on Health and the Roma Community lists factors that explain the poor health conditions of many European Roma citizens; among them are a lack of resources, poor housing conditions, inadequate employment opportunities, and difficulty gaining an appropriate level of education. The Roma also have inadequate access to prevention services: over 25% of Roma children do not adhere to vaccination schedules and 40% of women over 15 have never been to a gynecologist for reasons other than pregnancy or labor [22]. 

In contrast, particular cultural traits among the Roma can function as health assets. Among other traits, solidarity within the community, strong family bonds, and cultural norms and traditions surrounding health might help explain—and improve—the health conditions of the Roma people [24,25]. 

There is a lack of quantitative data specifically associating the health statuses of the Roma with their educational levels. However, the general association between low educational level and poor health status is especially threatening in their case, as the rates of exclusion that the Roma face in education, among other areas, are particularly alarming. One study that focused on the Roma population over age 15 in a selection of European countries with significant percentages of Roma people (Bulgaria, Czech Republic, Greece, Portugal, Romania, Slovakia, and Spain) found that 44% of Roma individuals had not completed primary school, and only 24% had at least a secondary education. Moreover, young Roma have a higher prevalence of risk factors for certain diseases linked to lifestyle and low educational status [26]. Thus, educational factors are recognized as having a significant impact on basic health indicators such as perceived health status. One of the few sources providing data about health conditions and education among the Roma is the report on *The health situation of the Roma communities* [27], which indicates, for instance, that 15% of Roma participants over age 14 who have finished post-secondary school suffer from chronic diseases, compared to twice that many (32%), among those who have no formal education. 

Researchers have found that, among other social factors, reduced inequality correlates with better health status and life expectancy [28], which is essential to overcoming the alarming health conditions in Roma communities [29]. In this context, studies have revealed the need to more strongly emphasize social inequalities [30] and to promote multi-sectoral policies (including health) that aim to reduce poverty by improving educational levels [31] to reduce the health disparities among the Roma communities and other ethnic minorities and vulnerable groups. 

In this regard, the academic community, along with the Council of the European Union and the European Commission, emphasize that substantial and effective efforts must be made to reverse the educational exclusion facing many Roma people across Europe to help reduce the health inequalities they experience [17,32].

### 1.2. Health Condition and Educational Level

Not surprisingly, people’s health improves when efforts are made to improve healthcare systems and to address other socioeconomic factors that have impacts on health, particularly education [33]. Indeed, the European Commission and other institutions highlight education as one of the four pillars of the social and economic integration of the Roma; they describe the potential of education to improve health and the impacts that health-related behaviors have on educational attainment [17]. 

Studies addressing the role of education in improving health have analyzed this relationship from the perspective of the educational level attained [11,16,34,35,36]. Given this focus, studies tend to neglect other important educational interventions or activities that could influence health. Although opportunities for lifelong learning have been recognized as helping to determine individuals’ health conditions [37], no systematic analysis has been conducted on how adult educational interventions could impact adults’ health conditions in OECD countries [38]. This research gap, in turn, indicates a potential gap in the way education interventions are analyzed: do they consider the relationships between education and health/wellbeing?

Indeed, it is widely accepted that education can directly influence health: greater knowledge contributes to better health. The influence might also be indirect: better jobs and higher incomes promote better health. These potential relationships are described in the WHO *Levelling up* report on European strategies for tackling social inequalities in health [37]. This report suggests that other educational elements might also play key roles in reducing health hazards, but those elements cannot be identified in analyses that focus exclusively on individual educational levels. That is, although the literature clearly shows the relationship between education and health quality, other factors related to involvement in educational activities might also be influencing those health improvements. For instance, a study on ways to overcome educational inequalities found that active participation and community engagement in decision-making in schools can help reduce health-related problems [39]. 

For all these reasons, then, further research is needed on the associations between education and health outcomes, especially accounting for groups at various socioeconomic levels [38]. Such research could reveal educational actions that can improve the health of particular vulnerable groups with low levels of education such as the Roma community addressed here. In this article we address this need and explore the particular ways in which two *successful actions* (SAs) that are focused on encouraging Roma people to participate in their local schools are improving health statuses. The integrated INCLUD-ED project (2006–2011) defined SAs as evidence-based actions in various social areas (education, employment, health, housing, and social and political participation) that are universal and transferable, and have been effective in addressing social inequalities and fostering social cohesion [40].

## 2. Methods

### 2.1. Data Collection

The analyses presented here stem from the INCLUD-ED Integrated project (2006–2011), the largest research project on schooling funded by the 6th European Commission’s Framework Programmes for Research [40]. The project’s general goal was to identify educational strategies that either contribute to social cohesion or lead to social exclusion. To this end, the project conducted a comprehensive and integrated analysis of European school systems, educational reforms, and educational practices that increase educational success. The project also explored the impacts that educational inclusion has on other areas of society such as employment, housing, health and political participation. The study was designed to focus on specific vulnerable groups (women, youth, migrants, cultural groups, people with disabilities, and ethnic minorities) to analyze the actions and policies that could promote their inclusion through education. 

In the context of this larger project, 26 case-studies of successful schools were carried out in different European countries. To select the cases, starting with data available from governmental sources, we selected those that most closely met three main criteria: schools that (1) were located in low SES areas with a significant presence of the vulnerable groups defined within this project, (2) obtained better educational results on standardized tests than average schools in similar contexts and (3) had high levels of family participation. 

The findings we present here are part of a four-year longitudinal case study [39] of a school located in La Milagrosa, a ghetto neighbourhood in the city of Albacete in southern Spain with a majority Roma population. This neighborhood is characterized by high levels of poverty; data from the Spanish Ministry of Education, Social Policy and Sports reflect that, in 2008, 35% of the inhabitants of this area were social welfare recipients, 7% were illiterate and 79% had not completed elementary levels of education. 

All of the INCLUD-ED research, including this case study, was carried out through Communicative Methodology [41], which is based on thinking from contemporary social scientists, including the communicative rationality as understood by Habermas [42] and the process by which humans construct knowledge through dialogue, which Mead calls symbolic interactionism [43]. This methodology was developed to create the conditions for egalitarian dialogue between researchers and the individuals they are studying; such dialogue breaks with the traditional interpretative hierarchy. This dialogue makes it possible to integrate existing knowledge from the academic community with the life worlds of the social actors using a communicative process that continues throughout the research: from the design of the research to the interpretation of the final results [44]. The new knowledge created through this process is relevant to the actors involved and, thus, is more useful for throughout society. This approach is especially valuable in research with vulnerable communities such as the Roma people, whose voices have traditionally been excluded from academic research, preventing studies from having a more relevant and useful impact [45].

To conduct the longitudinal case study (2007 to 2011) the project team collected both quantitative and qualitative data. More specifically, each yearly round included 13 standardized open-ended interviews (five with representatives of the local administration, five with representatives of other community organisations involved in the local project, and three with professionals working on the local project); 13 communicative daily life stories to end-users (six to family members and seven to students); one communicative focus group with professionals working in the local project; five communicative observations, and two questionnaires completed by the end-users (one addressed to family members and the other to students). 

In particular, the data from the fourth round (year 2011), which are analyzed here, consisted of 19 communicative daily life stories (communicative daily life stories are accounts given by research subjects in a cooperative process with the researcher. In this study, the interviewees were Roma adults involved in educational activities since the goal in collecting CDLS is to generate a shared understanding of the reality being analyzed through an egalitarian dialogue between the researcher and the subject of the research; the researcher contributes with knowledge from the academic community about the situation reality and the interviewee reflects on this information and contributes his or her own account of his or her daily life experience and knowledge of the given situation. This account includes critical events from both the past and the present.) conducted with Roma people, (among the communicative daily life stories conducted with the Roma, three were conducted with *mercheros*, people who, despite having lived among and like the Roma, are not considered by many Roma as being similar to them.) along with nine open-ended interviews (three with representatives of the local administration; three with representatives of community organisations and three with professionals working on the local project). As in most qualitative works, the participants were selected following the criterion of purposeful sampling [46], selecting the cases according to the stratification based on the previous knowledge in order to select those cases which could help better to respond to the research question. These techniques were the most appropriate for this research since the aim was the comprehension of the meanings that actors gave to their actions, and how they transferred the abilities and knowledge acquired through their involvement in the educational field to other contexts of their daily life, such as health and care. The research followed the criterion of “credibility”, “transferability”, “dependability” and “confirmability” which, as has been widely argued by qualitative methodology experts, are the base to ensure the validity of the results [47,48].

The data gathered in the phase presented here relates to self-reported health perceptions, which according to literature, is a good predictor of objective health status [49,50,51], as well as qualitative perceptions of education professionals and the other community representatives interviewed. Nevertheless future research could benefit from triangulation of this information with other sources, such as measures obtained from health records and qualitative research conducted with professionals of the primary care unit of the area. 

The voices of the Roma families and members of the La Milagrosa community are central to this study. Most of these participants have low incomes and little education beyond the primary level. They are all neighbors in the La Paz school community, and when they first became involved in the school, some were literate. These Roma adults had dropped out of school many years earlier, if they had attended at all. Additionally, few had jobs in the formal labor market, and they faced serious economic challenges, including how to feed their children. The community had experienced such a critical level of social exclusion that some had become involved in the vicious circle of drug abuse and dealing among other illegal activities. However, the transformation of the school into a Learning Community improved the situation of the whole community in a range of very important ways. 

The INCLUD-ED project was based on an analysis of *Successful Educational Actions (SEAs),* which are defined as the actions that contribute to school success (as reflected by students’ progress in educational attainment) and living together. In other words, SEAs contribute to overcoming school failure and early dropout, as well as overcoming the risk of exclusion in other areas such as employment, health, housing, and political participation. The SEAs identified have been shown to lead to positive progress in the results in every context in which where they have been implemented and therefore they have already been transferred to other schools and communities to improve school success and social cohesion.

Success is measured in terms of reducing early school dropout and absenteeism as well as in terms of the results obtained by students on the standardized tests administered by the Department of Education of Castilla La Mancha, which evaluates ten competencies, including, linguistic communication, mathematics, knowledge and interaction with the physical world, cultural and artistic competences, treatment of information and digital competence, social studies and citizenship, learning to learn, autonomy and personal initiative, emotional competences, and English. 

### 2.2. Data Analysis: Using Communicative Methodology to Include the Voice of Roma End-Users

The research team collected qualitative data so that it was possible to identify and analyze the impacts that the SAs had on students’ educational levels and on the health conditions of those participating in the activity and of others in the wider community. In this article it is analysed one SA, *educative participation*, which is described below, and its impact on the health of the Roma community in this neighbourhood. The voices of the end-users are presented here to explore the relationships between education and health that developed among this Roma community. We look at how, and how much, educative participation helps improve the health of Roma people. The accounts analysed here have been selected because they illustrate well the analytical themes that represent the findings discussed. 

The use of the Communicative Methodology was critical in both developing the data collection process and conducting the analysis. The communicative approach requires that the research subjects participate throughout the entire project. Various elements guaranteed this participation. One such element was the Advisory Committee, comprising members of the social group that is the subject of the research—in this case, representatives of the Roma community, and has the functions to provide feedback at different stages of the research on various materials, to analyze the literature review, to define the fieldwork techniques so that they would tap as much community knowledge as possible, and to review the final report, highlighting the evidence gathered. 

The analysis revolved around two dimensions, namely, the exclusionary and transformative dimensions. In analyzing a given situation of exclusion, those using the Communicative Methodology identify the obstacles to reversing the situation (the exclusionary dimension), along with elements that could help to promote change (transformative dimension). The transformative elements are key parts of this methodology; recommendations for ways to transform the situation can draw from both the existing evidence and the reflections and contributions of those who are experiencing the exclusion. 

## 3. Results and Discussion

The INCLUD-ED Integrated project identified several SAs in different domains that are helping reverse the social exclusion of vulnerable groups. Importantly; the results of this project have also informed several recommendations and communications approved by the European Parliament; the European Commission and the Council of Europe such as the motion for a European Parliament resolution on the EU strategy for Roma inclusion (and others) (European Parliament (2011). *Report on the EU Strategy on Roma Inclusion*; (2010/2276(INI)). Committee on Civil Liberties; Justice and Home Affairs; European Parliament (2009). *Resolution of 2 April 2009 on educating the children of migrants;* (P6_TA-PROV(2009)0202). (Brussels); Council of the European Union (2010*). Council conclusions of 11 May 2010 on the social dimension of education and training;* (2010/C 135/02) (Brussels); European Commission (2011). *Tackling early school leaving: A key contribution to the Europe 2020 Agenda;* (COM(2011)18) (Brussels); European Commission (2011). *Proposal for a Council Recommendation on policies to reduce early school leaving;* (COM(2011) 19) (Brussels)). This article describes the ways in which family and community educative participation at La Paz school had impacts on improving the Roma end-users’ health. Overall, we found that the participation of Roma families and community members in Interactive groups and Family education, both identified as *SEAs* [52], fostered specific cultural values among the Roma that support better health. Moreover; they transferred some of their learning in the SEAs to other contexts. Both elements helped to improve not only their own and their families’ health conditions but also the general level of health in their community. 

### 3.1. Roma Cultural Values as Assets: Improving Health through Educative Participation

Two cultural traits of the Roma—family cohesion, and mutual care and protection—play key roles in their health conditions. For the Roma, the value of family includes great respect for the elderly and authority, care for one’s family members, and the observance of cultural family norms such as those related to mourning and kinship solidarity [53]. Importantly, the Roma understand family not as the nuclear family, but rather as the extended family and even the broader community. As they organize their lives, they include all people whom they consider to be near their kin, and whom they identify with their homes, their culture, and their way of doing things. Thus care and relationships also extend to a wider idea of family, more similar to that of community. This conceptualization of community or extended family is illustrated by Aroa, a 25-year-old Roma woman who has siblings and cousins in La Paz school. She explained in her narrative that “everybody knows each other in the neighbourhood and if they are not cousins, they are nephews... we are all relatives here”. 

Thus, the Roma people have a strong sense of identity and belonging to the broad and diverse Roma ethnic group, they share identity traits, such as the family and community-based ways of organizing, the networks of solidarity established within these groups, and the value attached to dialogue and the word [54]. All of these values arise from their longstanding experiences of discrimination. Their centuries-long opposition to injustice has become a unifying force instead of a divisive one, contributing to their cohesion and survival as an ethnic group. All of these experiences have led the Roma people to be aware of and care about not only their personal wellbeing or that of their closest family but also that of the other members of the community, regardless of others’ actual roles as kin. This sense of common identity motivates the strong bonds of solidarity that the Roma develop among themselves, a factor that can also help to improve their health. This trait is clearly illustrated in the words of Luis, a 42-year-old Roma father in the neighborhood:

*“If we Roma can be identified by something it is by this unity with this strength [as people], even more so, we are there in the moments of illness, 100%. For instance, if someone needs blood, and if I [meaning the community] can’t donate, we buy it somewhere and we provide it. And we ask each other if [my blood] is good or if yours is, and if we are 100 people, the whole 100 are tested to save the one person. This is a point that I believe identifies us very much, very, very much”.*

The analysis of the ways that Roma families and community members participated in Interactive groups and Family education at La Paz school showed that this involvement helped these families to gain access to more resources and promoted relevant skills among them; moreover, it enhanced particular Roma cultural strategies and values that helped them extend these health improvements to the whole community. The next sections explore in more depth how their educative participation helped reduce some of the health inequalities in the community and therefore enhanced their health overall. 

The INCLUD-ED project identified educative participation as one of the three types of participation that had the greatest impacts on children’s academic achievement [55]. Educative participation means that members of the students’ families, as well as others in the community, either become involved in instrumental learning activities with the children or participate in education programs themselves. Among these activities, Interactive groups and Family education were considered SEAs because they helped improve children’s academic outcomes and were transferable. The analysis of the educative participation of Roma families and community members at La Paz school showed that this involvement enhanced particular Roma cultural strategies and values that helped them extend health improvements to the whole community.

Interactive groups have been found to be a successful form of heterogeneous ability grouping and reorganization of human resources within the classroom; such groups allow all the students to perform better academically, and they improve coexistence. When a teacher implements interactive groups, the classroom is organized into small and heterogeneous groups of students, each with an adult (a teacher and/or a volunteer) who promotes supportive interactions and dialogue among the students. The classroom teacher manages the classroom and provides extra support when necessary [52]. In this strategy, family members or neighbors come into the classroom to support the teacher and participate in the children’s learning. Family education activities are addressed to adults and include literacy, subjects that are part of basic primary and secondary education, and a wide set of courses that train individuals for work in such areas as childcare, care of the elderly, and monitoring playgrounds and school cafeterias. 

**Figure 1:**
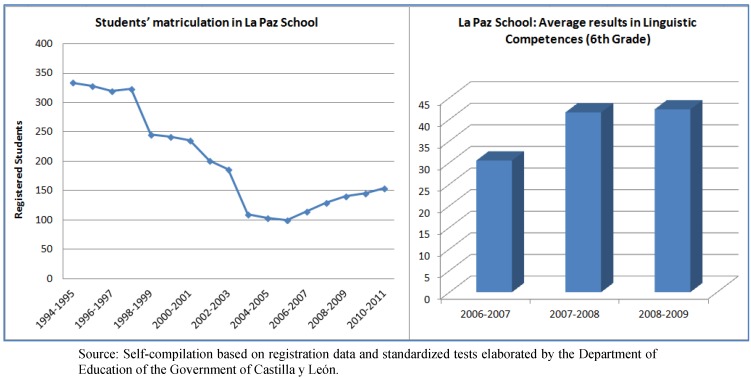
Pupils’ matriculation and results in linguistic competences in La Paz school.

The implementation of Interactive groups and Family education in La Paz started in the 2007–2008 school year. Educational results have improved since its initial implementation, according to the Diagnostic Tests conducted by the regional government of Castilla La Mancha in all schools. As is shown in Figure 1, the variations in the pupils’ matriculation provide further evidence of school success. Moreover, absenteeism for the 2006–2007 school year was 30% and it reduced to 10% the following school year. 

When members of families and the community come into the La Paz classrooms that are organized into interactive groups, they are in charge of encouraging supportive interactions among the children. In addition, through the family education in La Paz, the Roma people acquire instrumental competences (*i.e.*, in languages, math, or science); this also affects their children’s success in school [52,56,57,58]. In this regard, the evidence has long shown that family involvement in educational activities has a positive impact on children’s academic performance; it changes their school-related behaviors and improves their achievement. Thus, family education enriches interactions among those of different cultural and educational backgrounds, making that diversity an asset that can further improve both academic results and the coexistence among children of different backgrounds. Among the other impacts are the increased interactions between family members and the school as family members follow up on or ask for help with the child’s school activities. Through both interactive groups and family education, Roma parents become active agents in their children’s learning; in turn they feel increasingly useful, self-confident, and rewarded for their work [59]. Such participation also helps to create networks of trust among participants which later translate into increased trust in the use of various social services, such as health care [60]. The focus here is how such family and community participation in schools can become a space to support health improvements among the Roma.

### 3.2. Increased Emotional Wellbeing through Educative Participation

“I feel much happier, much more satisfied and even more valued… because I wake up with a purpose”. Luis’ initial involvement at the school was not his own decision. He started volunteering at his children’s school while he was still in prison to comply with the requirement for community service, however, his participation in Interactive groups at the school radically changed his life. When he was released, he offered to continue as a volunteer at the school.

Luis’ experience is like that of many Romani parents at La Paz. As the school opened to the community through its process of becoming a Learning Community, many opportunities became available for the local Roma people who had, until then, lived at the margins of society. Luis did not simply participate in the classroom; in the Interactive groups he became a relevant resource in several ways, improving the learning processes of all of the children in the community, as well as his own. His participation in appropriate educational activities, such as the interactive groups, made him feel valuable and useful. The Roma people were interviewed agreed on this point: as soon as they became involved in the school’s daily educational activities, they became happier and more motivated, and active in the regular activities of their daily lives. In addition, other people observed Luis’ improvement, including the teachers, his relatives, and the children. Luis explained,

*“To be appreciated by someone is nice, to be appreciated by many people and children, and not only children but teachers, even though the appreciation of the children is important, too (…) because before you got up without a purpose. Now you get up with another, you know, with another perspective...”.*

New routines, habits, resources, and even sources of motivation have all improved the lives of these Roma participants and are directly impacting their personalities, ways of thinking, expectations for their future and that of their families, and ways of communicating. These attributes also affect their health and wellbeing, as described by Luis:

*“Well, as I said, it is because of coming here, as I said, the way of talking, the way of thinking, the way of relating to another person (…) Now I have a rhythm, I come in the morning, in the evening, but I truly like it”.*

For these Roma parents, the very act of participating in activities in which they see their children learning becomes a trigger that increases their emotional wellbeing; it creates meaning that improves their self-esteem. Rosario, a Roma mother of six children who was in jail for dealing drugs, explained this when talking about her involvement in interactive groups:

*“You could see how children learned, how useful you felt… teachers ask your opinion. In the classrooms you are like if you were also a teacher; they ask you to do something with the children, and then you feel useful, you feel well, you feel valued. And now even more!”.*

They also see that being involved in activities in which they help future generations learn improves their own self-esteem, because they feel useful and see themselves doing something good for others, —in contrast with some of their past personal experience. In this vein, when discussing the impact of her involvement in the school’s educational activities, Rosario explained that it had helped her as well as the children. Now she feels that she is a better and more useful person:

*“It has helped me a lot! Because in addition to helping my children, I am also learning, as it’s stuff that you have forgotten and also I personally feel very useful and I feel I am a better person when I come here...”.*

This involvement by Roma adults in the interactive groups or family education activities has given new meaning to their daily lives. They now do something that they know is positive, both for themselves and for others in the neighborhood, and this leads them to see more worth in themselves. For many in La Milagrosa, these motivations to collaborate have helped to prevent mental illness in the forms of depression and drug or alcohol abuse. 

### 3.3. Raising Awareness about Health Issues

In addition to feeling more motivated and important in others’ lives, these Roma adults also indicated that participating in the school’s educational activities made them more aware of health problems in the community and the need to intervene. When they are involved in the classroom, the mothers become more aware of health conditions among their own children and the other children, leading them to improve their own attitudes about health. The teachers especially noticed this impact, as in the example below; Monica, a teacher from La Paz school, explained how family members’ participation in interactive groups had an impact on hygiene conditions:

*“In my class, yes I do, because [the mothers] see it. They see how many children come in very clean, and then theirs...you know? (…) So yes, they themselves notice. For instance one came and said: check their heads because there’s lice …and when they are in class participating, they see this even more”.*

Thus, the educative participation of these Roma adults has made them more aware of health-related issues such as hygiene, along with auditory, visual, and dental problems among the children. 

Similarly, people interacting within the interactive groups and family education environments constantly raise and discuss various issues related to children’s health. Now, Roma family members can acquire important information that they did not have before; they also learn new strategies and resources to improve the community’s health. For instance, they discuss children’s vaccinations and ways to prevent and treat head lice, and they share information on dental and eye care during informal interactions with teachers, students, and other family and community members. 

Thus, family participants learn about health-related topics while they acquire new instrumental competences in various other fields, including math, languages, and science. Perceiving themselves as learners, they adopt a different attitude as they hear details about health care or illness, becoming more interested and more active agents in both the educational arena and their families’ health. Now, when they go to the doctor they do not simply comply with instructions; they want to know and understand what the terms mean and why the doctor prescribes one treatment over another. Luis explained:

*“I go to the doctor, or to my daughter’s doctor, and I get information with all the details; if there is anything that I don’t understand well, they’ll have to explain it well, because I’m interested in it (…) For example, I might not know a particular disease, or they would say, ‘you have to go to the otorhinolaryngologist’, and I would have said ‘yes, yes…’; I was ashamed to ask, and I’d said yes, and then once I was in the street I would tell someone that they had told me to go to the ‘otolary’ something, and they would say ‘but will you go there?’ and I’d be ‘but where?’ And now maybe I say ‘ear, throat and nose specialist’ because they have explained it to me because I have asked about it. I have asked him, ‘and what is that about, what is it for, what function does it have?’ And I don’t feel ashamed to ask.”*

In sum, the data analysis demonstrates the ways in which educative participation has a positive impact on the Roma health, both because individuals acquire new skills and competencies and because they have generated more confidence in this new learning environment. 

### 3.4. Caring for the Family’s Health

Dolores is 26 years old and is participating in a family education course to earn her certificate in secondary education. She explained that since she became involved in the school, she has paid more attention to the health of her four younger brothers. She dropped out of school when her youngest brother was born; her mother fell into a deep depression, and Dolores had to take care of both her mother and brother and assume the household tasks. Now, as she takes care of the family, she establishes healthy routines and habits, which she sees as her responsibility.

When Dolores began to participate at La Paz, she maintained her family responsibilities but still engaged in other activities that benefited both her and her relatives. Other Roma women told similar stories. Their domestic routines changed as soon as they became engaged at the school; now, they can better organize their everyday lives to make optimal use of their time. They participate at the school, assume family responsibilities, and also take good care of themselves.

*“There must be time for everything: to get ready, to take care of oneself, to feel well… and not be the typical Roma with the bun and the slippers and the housecoat... no, that has to change! That was in earlier times, in the first century, in the second century… one must become independent and go ahead on your own, as well (…) I used to say that women had to be at home, washing the windows and staying with the kids and nothing else. But look at me four months ago… I don’t recognize her! And some tell me, ‘you used to say that!’ and I [respond], ‘well, I used to say that last year, but this is already a new year!’ And now Dolores is not the same anymore. One has to update!”*

Importantly, these transformations show the positive impact on families’ health qualities. These women take home all that they learn through their new interactions at the school. Dolores talks about her family’s nutritional habits, especially those of her younger brothers, to whom she is now much more attentive:

*[In a joking way, talking about healthy eating habits] “I’m an artist, in that I’m worse than my mother! The kids are disgusted by me because of the vegetables, and I say, “mummy prepares boiled things, no fried stuff, because the kitchen ends up greasy and we don’t eat well.” [Does she really call herself mummy? She’s their sister!] “I try to get my brothers not to eat chocolate pastries. And now, in school, the same - vegetables, and now with the fruit, very well. (…) Milk? No, they want Nesquik [chocolate milk]. Well, none of that! ‘You’ll eat what we have. Don’t ask me for funny things, I’m not going to buy them’… I try, I try with the vegetables.”*

As the adults spend time in the educational environment, hear of issues related to health, and acquire important new skills and competencies, they begin to detect and prevent their own health problems along with those of family members. In addition, they bring the knowledge that they acquire in the school to other areas of their lives, particularly their homes. The importance of family care and cohesion among the Roma, along with communication and an emphasis on dialogue, helps to reinforce the learning that takes place at the school. Other members of the community know that if they need help with or advice about personal or health issues, they can rely on support from those who participate in the school. For instance, Aroa, who is becoming more literate through her participation in La Paz, explained how she helped her aunt, who is still illiterate, to understand what the doctor said in a letter to her:

*“[My aunt said] ‘I have received a letter from this person.’ Most of my aunts can’t read or write, so she told me, ‘the kid read it for me, and I have to go and see. You come read it for me, let’s see what it is.’ ‘Well,’ [I told her] ‘you have to go and have this test done and so...’ and [she asked] ‘what’s that for?’... of course they ask you... ‘did they find something wrong with me?’ because they are like that, because they are uneducated,… [and I said] ‘it’s just in case, nothing’s gonna happen, but it is good to have the test done.’”*

As seen here, the strong family bonds among the extended Roma become a channel through which information flows from the person involved at the school to the rest of the family, including those who have no children at the school, such as grandparents. The emphasis on family wellbeing and the relevance of dialogue and communication ensure that what one person learns will flow to others in the household. Given this phenomenon, the conversational topics within the family change with time, and they expand to include health concerns. Luis explains how this occurred in his family:

*“Well, I just go there and tell them everything, from an anecdote about some child to the discussions with them, all around the clock, from when I get up until I fall asleep...it is all talking about school. Besides, I like to tell my wife, my children… while we eat I explain to them, ‘this and that has happened to me’. It’s a joy at home… we used to communicate as well before, we talked but it was more like there was nothing to talk about...”*

Family education courses in La Paz serve two purposes. First, the Roma participants acquire literacy skills and other competencies that they can use to capitalize upon their existing knowledge, gaining skills, for instance, in assertiveness and communication to more effectively respond to doctors’ instructions or the pamphlets that come with medications. Second, they transfer their newly acquired knowledge about health issues to their families, where others then use it to make more appropriate decisions about their own health. The following quote from Luis illustrates how, within an adult education course, participants also obtain access to specialized health knowledge, which has a positive impact on their families:

*“Even the podiatrist has come to the adult learning school (…) because maybe you think that you need to get surgery on your foot, and then this man tells you that your shoes are not right, and maybe you don’t need any surgery (…) Well, this man told us that we have to walk like this and that, and I went to see my kids and I told my daughter ‘walk, walk,’ and I said ‘you have to walk like this,’ as he showed us, about the movement, and so on (…) And after seeing the podiatrist, I came home and I said ‘let’s see whether my daughter has this curve to her feet,’ but if you don’t have this information… well, then that’s it.”*

Along the same lines, Rosario, who is involved in interactive groups and family education in La Paz, explained how she is taking better care of her children; she says the same is happening in other families and that people are changing in deeply transformative ways. Her involvement even helped her to overcome her addiction to drugs, which led to deep changes in her own life and those of her family members:

*“By being involved in school... for instance, there are mothers whose children came to school very scruffy, and by coming here [to the interactive groups], you change your image completely. You take better care of them. This has happened to me. There was a time when I was very bad, I was on drugs. I was in a center for nearly seven months. And at that time I did not take care of my children. On the contrary, they got up on their own, got dressed, and came [to school]. Later on, when I left the center and I was well, it was me and my husband. And I have not fallen back into drugs, thanks to the school, coming to school, getting involved... for me, it is very important, very important.”*

Overall, through such involvement, these Roma people have begun to care more about their own health conditions and those of their families. Their participation has deeply transformed their lives and future prospects at different levels. Not only do they capitalize on the knowledge they acquire and share it to benefit their families; with transformed attitudes and increased confidence, they can also better address health issues and develop more informed relationships with healthcare staff. Their particular cultural values, with the prevailing relevance of family traditions and norms, play significant roles as they improve their own health-related habits and those of their family members. Importantly, these cultural values help to extend the benefits of this participation beyond themselves to their families and even beyond their closest kin, as shown in the following section. 

### 3.5. Caring for the Health of the Extended Roma Community

Because interactive groups and family education emphasize dialogue among all participants, they can take optimal advantage of the elements of Roma culture, such as unity and group cohesion, which, in turn, improves Roma health. For instance, when one Roma adult learns that any child in the school has a health problem, she or he reacts quickly, talking with the child’s parents or other relatives to find a solution that will improve the child’s wellbeing. For example, because of her involvement in an interactive group, Aroa was able to identify why Miriam, a Roma girl in the classroom, was nervous and fidgety every morning. Aroa observed Miriam’s behavior and asked her about it:

*“Why are you so nervous? And Miriam answered, ‘because I don’t have chocolate milk for breakfast. My grandmother gives me coffee because she doesn’t have chocolate milk.’ So then… I will, of course, it’s the girl’s health; she is just 8 or 9 years old and drinking coffee (…) The grandmother was misinformed...”*

When Aroa came to understand why this girl was so nervous, she spoke with the mother about the problem she had noticed. As a member of the Roma community, Aroa shared more cultural codes with the mother than the teacher did, so she and the teacher decided that she, rather than a school employee, would raise the issue with the mother. In talking with the mother, Aroa was able to inquire more about the girl’s health; she then learned that Miriam’s younger sister, only five years old, also had this unsettled behavior because of her inappropriate breakfast. Aroa’s intervention was invaluable; it led to both girls joining the Morning Class, a school initiative in which children from families in need receive healthy breakfasts before starting the school day. Aroa’s account vividly exemplifies several relevant elements grounded in Roma culture that are positively associated with improving health conditions and overall care among the Roma community. We see her concern about the fidgety girl and how she addresses the issue with both the girl and her mother through open and confident communication. 

This Roma commitment to the health of others in the community serves to increase the trust among other Roma parents who are less involved in the school; they feel relieved that their children are taken care of by the women who volunteer there. Aroa expressed that she could see that other Roma participants were also committed to the wellbeing of other children in the neighborhood. She also realized that even though she had no children of her own in the school, she was more concerned with the other children’s health and wellbeing because of her involvement in interactive groups and family education:

*[About health] “I am more interested… I remember it and I see it. When I arrive at the school, I see that they are also interested in the health of children. I do not have children myself, but I have other relatives in school, and I see that and whether I want it or not, I also get it there too, it’s transmitted. I mean, I also want to be interested in them, being family, mother or whatever.”*

These mechanisms have allowed the Roma people to transfer their new knowledge and skills about health beyond the school and the family to the rest of their community. The experience of increased solidarity among various community members enhances relationships among all the neighbors, as they encourage each other to adopt new positive habits. Juana, another Roma mother, explained how the environment had become friendlier as families in the neighborhood cared more for each other:

*“Yes, yes, there are fewer conflicts. Yes, because the mothers come to the school, they maybe have a coffee, become more friendly, trust each other more, and when you are on the street you say, ‘hi, so-and-so.’ Maybe before we didn’t say ‘hi’ to each other and now we do, and ‘are you going to school, or have you gone?’ or ‘why didn’t you go today?’ This stuff.”*

In addition, this positive social control has extraordinary consequences for some of the participants, as solidarity and community care have led them to engage more deeply. Vanessa, a local teacher, explains another side of Rosario’s story; her life changed through her participation in the interactive groups, encouraged by community members and the teaching staff. This change deeply affected her health habits and routines as well as those of her family:

*“And, well, we kept on encouraging her, saying, ‘come on, just come one day and try, if you like it, you can offer your ideas’… and from the moment she came, she liked it, and she keeps on coming. Everything improved. In school, her kid is much better, both in learning and behavior. At home, she has started to organize herself, her house is now cleaner (…). Now she has habits and routines that allow her children to get around and look for work. She is also pursuing training herself, getting involved in the education of her children and taking more care of everything. One thing led to another…”*

These women’s stories show the deep changes in their lives as a result of their involvement in the interactive groups and family education, which then led to changes in their families and the community. Indeed, they recommend that other Roma become involved at La Paz as an effective way to improve their children’s futures as well as their own circumstances and their families’ health. For example, when a friend of Aroa’s was depressed, Aroa urged her to become involved:

*“A girlfriend of mine has had deep depression, and I have tried to encourage her to take a course [in the school] or be a volunteer in the school, to keep her mind busy with something meaningful.”*

Also increasing the impact on the community is the fact that a wide variety of Roma adults from the neighborhood are involved in the interactive groups and family education. When health issues arise, they are granted a kind of authority among Roma children and youth that even the teachers do not hold. This authority, another cultural element, also helps to improve health habits. The teachers understand how this can be an asset and use it to improve the health of all the children. Luis explains this clearly:

*“They caught one or two kids in the schoolyard with a cigarette, and they said to me, ‘look, Luis, what if you talk to them, as a Roma and as a man? They will not shout at you or swear at you’. So I did talk to them (…) Yes, yes, if it had been the teacher [female] they would’ve told her whatever, but if it’s me, there’s almost no need to say a word. They would get quiet, no swear words, no yelling, nothing.”*

This vignette illustrates yet another finding: by spending more time in the school with the children, the Roma adults become able to identify problems among the children that their families often do not notice or cannot control. The cultural emphasis on community members as authorities increases the value of having Roma adults present within the school; they can exert positive social control while reinforcing the bonds of trust among Roma boys and girls and the Roma adults. 

## 4. Conclusions

Researchers have taken individuals’ educational levels as positive indicators of their health conditions, assuming that people with no or little education are less likely to be healthy [16,61]. This association makes even more sense for vulnerable groups such as the Roma, who receive far less formal education, on average, than the mainstream population [26].

The results that have been presented here indicate that educative participation is a successful action (SA) that has a positive impact on the health conditions of Roma people with little education. The analysis of this Roma community has shown that participating in such SAs can lead to improved health, even among people as vulnerable as the Roma. 

Moreover, we found that the positive impact on health by involvement in these activities transfers to other domains, including the prevention and early identification of mental illnesses, such as depression and anxiety, and health issues arising in early childhood, such as those related to hygiene, dental health, and hearing, among many others. Additionally, participating in these SAs can help people acknowledge and overcome drug abuse. 

The participants provided a wide range of reasons to explain this improvement. Some of these reasons are applicable to other social groups; for example, nearly anyone will feel more vitality and motivation for living when they engage in a meaningful and socially useful activity. However, given the extreme health inequality facing the Roma, our focus here has been on the explanations that are most applicable to this ethnic group. The Roma participants spoke of strategies relevant to their cultural tradition that were enhanced by their educative participation. The experiences of Aroa, Luis, Rosario, Dolores, and Juana illustrate how Roma people, even those with little formal education, can build on particular cultural norms and traditions that are supported in the context of this SA to improve their health-promoting habits. 

When Roma participants in the school feel that the children’s health is also their concern and responsibility, they become even more involved in their own personal care and in identifying health problems, whether physical or psychological, that affect their children or other people’s children. In doing so, they gain knowledge and resources about health that others in the community may not have; however, because the interactive groups and family education foster family and community communication, which are also key Roma values, these Roma adults transfer their new knowledge to others in the community. We also found that when these people faced new health problems, the solutions they generated were based on their own cultural values and strategies, such as involving members of the extended family and engaging in dialogue with their children and families. Direct communication and open dialogue appear to be the core of community-based problem resolution among the Roma people due to the strongly rooted value within Roma culture that helps reduce health hazards and increase health-promoting behaviors. Both their deep emphasis on family cohesion and their strong sense of belonging to the same ethnic group are reinforced when Roma people participate in community-based learning spaces, such as interactive groups and family education. Participants in these SAs use strategies grounded in Roma cultural values, which ultimately benefit not only their own families but also the extended Roma community. 

Importantly, these two educational actions, interactive groups and family education, share two key principles that overlap well with the health improvements in the Roma community: (1) the goal of optimizing participants’ instrumental learning and (2) an emphasis on egalitarian dialogue, in which anyone is welcome to contribute to the dialogue, regardless of their social or academic status. The two principles work together to enrich the bonds of solidarity within the extended Roma family and beyond; in turn, this solidarity helps participants transfer what they learn about health in the school to other spaces, reaching the broader Roma community. 

Future research is needed in order to complement the data obtained. Particularly, it would be interesting to contrast the information obtained with available health records of the population of La Milagrosa as well as with fieldwork conducted with primary health professionals of the area.

Evidence that educative participation can effectively improve health conditions among the Roma people is a positive contribution to the goal to reduce the health inequalities from which they suffer. Thus, in addition to simply helping people gain more education, these other educational interventions can help them to overcome health inequalities. Furthermore, our findings emphasize the need for researchers to include the voices and cultural strengths of the Roma and other vulnerable cultural and ethnic groups when designing educational interventions that aim to improve their health conditions. Finally, policies intended to reverse the health inequalities facing the Roma people must be grounded in empirical findings, such as those provided by the Roma community in La Milagrosa in Albacete; in this community, people have transformed their health-related behaviors, regardless of their educational levels, through the implementation of evidence-based SAs. Future works addressed at the development of health policies for Roma will benefit from the findings of this research. 
